# Targeting lipogenesis in the treatment of metabolic diseases and cancer

**DOI:** 10.18632/oncotarget.23004

**Published:** 2017-12-06

**Authors:** Jose A. Viscarra, Hei Sook Sul

**Affiliations:** Hei Sook Sul: Department of Nutritional Sciences and Toxicology, University of California, Berkeley, CA, USA

**Keywords:** lipogenesis, MED17, CK2, USF1, FASN

Lipogenesis must be regulated tightly to meet metabolic needs according to nutritional status. During fasting, fatty acid synthesis is virtually absent mainly due to increased glucagon/cAMP signaling [1]. In contrast, during feeding, fatty acid synthesis mainly in liver increases drastically, especially when the diet is carbohydrate rich, as glucose utilization and glycolysis increase [2]. Increased levels of circulating glucose and insulin contribute to the induction of lipogenesis. Many enzymes involved in fatty acid and fat synthesis, including fatty acid synthase (FASN), ACC, SREBP1c, ACLY, and mitochondrial GPAT are upregulated at the transcriptional level in response to insulin signaling [3]. FASN plays a central role in de novo lipogenesis by catalyzing 7 reactions for palmitate synthesis from acetyl-CoA and malonyl-CoA, and is regulated at the transcriptional level during the fasting/feeding cycle [3]. Therefore, FASN makes an ideal target to study the transcriptional activation of lipogenesis.

In understanding the transcriptional activation of FASN and other lipogenic genes by feeding/insulin, we have focused our efforts on upstream stimulatory factor 1 (USF1) and the signaling pathways involved [3, 4, 5]. While there are several transcription factors, such as SREBP1c, LXR, and ChREBP that are known to promote lipogenic gene transcription, we found that USF1 plays a central role as its binding to the -65 E-box is required for the activation of the FASN promoter by feeding/insulin [3]. Furthermore, we have shown that multiple signaling pathways activated by insulin converge on USF1 and its coactivators in activating lipogenic genes during feeding/insulin treatment [3, 4]. Briefly, insulin causes PP1 translocation to the nucleus to dephosphorylate/activate DNA-PK that, in turn, phosphorylates S262 of USF1. This is the first time that DNA-PK was shown to be a downstream kinase in insulin signaling. Thus, after being phosphorylated by DNA-PK, USF1 interacts with P/CAF to be acetylated at K237. Phosphorylated/acetylated USF1 can then interact with BAF60c, which is itself phosphorylated at S247 by atypical PKC in response to insulin. The interaction between USF1 and BAF60c results in the recruitment of the LipoBAF complex to the FASN promoter for chromatin remodeling and transcriptional activation [3, 4].

**Figure 1:**
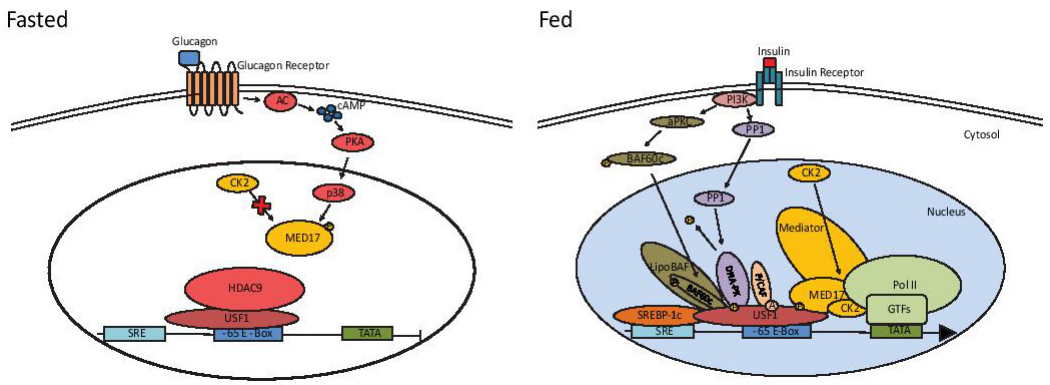
Post-translational modifications to USF1 during the fasting-feeding transition and their effect on lipogenic gene transcription During fasting, HDAC9 interacts with USF1 for its deacetylation, which blocks interaction with other cofactors. Signaling through the Glucagon-cAMP-PKA axis activates p38 to phosphorylate MED17 at T570, preventing its phosphorylation by CK2. During feeding, insulin activates PP1 to dephosphorylate DNA-PK, which then phosphorylates USF1 at S262. Phosphorylated USF1 interacts with P/CAF and is acetylated at K237. Phosphorylated/acetylated USF1 then interacts with BAF60c, which is phosphorylated by atypical PKC at S247, to recruit the LipoBAF complex for chromatin remodeling. In the absence of T570 phosphorylation by p38, MED17 is phosphorylated by CK2 at S53, and interacts with USF1 to recruit the Mediator complex and the general transcription factors to activate transcription of lipogenic genes.

Recently we identified Mediator complex subunit 17 (MED17) as a USF1 interacting protein which, by directly binding to USF1 recruits the Mediator complex to activate transcription following feeding/insulin treatment [5]. We determined that MED17 is phosphorylated at S53 by CK2 and that this phosphorylation is required for the activation of transcription of lipogenic genes by feeding/insulin. We also found that MED17 is phosphorylated by p38 MAPK at T570 in cultured cells in serum-starved condition, and that this phosphorylation event prevents subsequent phosphorylation by CK2, since a MED17 T570D mutant showed an impairment of MED17 phosphorylation at S53 [5]. This work identified an additional signaling pathway by which insulin activates lipogenic gene transcription (Figure 1). Moreover, we also found a potential means by which lipogenic gene transcription can be turned off. In order to examine potential dysregulation of these pathways in obesity, we assessed phosphorylation of MED17 in liver of leptin-deficient and insulin resistant, ob/ob mice and found chronic phosphorylation of S53 even in the fasted state [5]. This suggests that persistent phosphorylation of MED17 at S53 may contribute to the chronic activation of lipogenesis and to the onset of obesity and diabetes in ob/ob mice.

Dysregulation of lipogenesis has long been recognized as a contributing factor for multiple metabolic diseases including obesity, hepatosteatosis, and insulin resistance [2]. However, dysregulation of lipogenesis is observed often in cancer also, since the fast dividing cells have greater demands for lipid substrates for cancer cell proliferation and survival [6]. Interestingly, activation of SREBPs and their downstream targets have been implicated in cancer development [7]. Therefore, previous studies have targeted lipogenic pathways in developing therapeutic strategies for the treatment of cancer. Recent work has focused on the use of inhibitors against FASN or ACLY to block lipogenesis post-transcriptionally, or silencing ACC to induce apoptosis in cancer cells [6]. These strategies show some level of success and so provide evidence that targeting lipogenesis may be effective in the treatment of cancer. As stated earlier, we identified that phosphorylation of MED17 at S53 by CK2 is required for the transcriptional activation of lipogenesis. Furthermore, we showed that overexpression of MED17 (S53A), a nonphosphorylatable mutant, as well as CK2 inhibition significantly decreased lipogenesis *in vivo* [5]. CK2 inhibition using CX-4945 has previously been examined as a potential therapy for the treatment of cancer since its function as a pro-survival kinase makes it a prime target in stopping cancer cell proliferation [8]. However, CK2 is ubiquitously expressed having a large number of substrates in wide-ranging biological functions [8] and thus it is not clear whether chronic inhibition of CK2 is suitable in the treatment of metabolic diseases or cancer. Targeting MED17 directly may also be an option, but we need to understand whether CK2 effect on MED17 is specific to lipogenic genes, or if there are other targets, by examining at the global level.

In summary, our continuous work attempting to understand the complex transcriptional networks that regulate lipogenesis revealed yet another pathway critical for the transcriptional activation of lipogenic genes. Since dysregulation of lipogenesis is a contributing factor of many diseases, identifying therapeutic targets within lipogenic pathways is of utmost importance.
